# Orthogonal Activation of Metabotropic Glutamate Receptor Using Coordination Chemogenetics

**DOI:** 10.3389/fchem.2021.825669

**Published:** 2022-01-14

**Authors:** Akinobu Senoo, Yutaro Yamada, Kento Ojima, Tomohiro Doura, Itaru Hamachi, Shigeki Kiyonaka

**Affiliations:** ^1^ Department of Biomolecular Engineering, Graduate School of Engineering, Nagoya University, Nagoya, Japan; ^2^ Department of Synthetic Chemistry and Biological Chemistry, Graduate School of Engineering, Kyoto University, Kyoto, Japan; ^3^ ERATO (Exploratory Research for Advanced Technology, JST), Tokyo, Japan

**Keywords:** chemogenetics, coordination chemistry, orthogonal activation, class C GPCR, metabotropic glutamate receptor

## Abstract

Cell-surface receptors play a pivotal role as transducers of extracellular input. Although different cell types express the same receptor, the physiological roles of the receptor are highly dependent on cell type. To understand each role, tactics for cell-specific activation of the target receptor are in high demand. Herein, we developed an orthogonal activation method targeting metabotropic glutamate receptor 1 (mGlu1), a G-protein coupled receptor. In this method, direct activation *via* coordination-based chemogenetics (dA-CBC) was adopted, where activation of mGlu1 was artificially induced by a protein conformational change in response to the coordination of a metal ion or metal-ion complex. Our structure-based protein design and screening approach identified mGlu1 mutants that were directly activated by the coordination of Cu^2+^ or Zn^2+^, in addition to our previous Pd-complex-sensitive mGlu1 mutant. Notably, the activation of the mutants was mutually orthogonal, resulting in cell-type selective activation in a model system using HEK293 cells.

## Introduction

Several hundred types of receptors are expressed on the cell surfaces of mammals, each of which plays essential roles in transmitting extracellular information into cells. Moreover, the same receptor is found in different cells or tissues, yet the physiological roles of each different receptor are highly dependent on the cell or tissue type (Vassilatis et al., 2003). Selective activation of receptors of interest in cell lines or primary cell cultures is a useful way to investigate the physiological roles of these receptors. However, chemical methods alone are insufficient to analyze the roles of receptors in a cell-specific manner in particular tissues or organs because of the challenges associated with cell-specific delivery of chemicals (Mondoloni et al., 2019). Thus, the development of new tools for cell-specific activation of target receptors is highly desired.

Chemogenetics, a process in which proteins of interest (POIs) are genetically engineered to selectively interact with designed chemicals, is a potential approach for cell-specific activation of target receptors (Islam, 2015; Atasoy and Sternson, 2018; Tsai et al., 2021). Some representative examples of chemogenetics include the bump-and-hole approach (Bishop et al., 2000; Knight and Shokat, 2007), ligand-induced stabilization (*i.e.*, chemical rescue) (Pratt et al., 2007), and chemically induced dimerization (Schreiber, 1998). Another well-known example of chemogenetic activation of G-protein coupled receptors (GPCRs) is designer receptor exclusively activated by designer drugs (DREADD) (Armbruster et al., 2007; Urban and Roth, 2015). In DREADD, the designer receptor derived from muscarinic acetylcholine receptor is selectively activated by a designed chemical, clozapine-*N*-oxide, but not endogenous ligands. Similar approaches have been reported using other GPCRs such as the β2 adrenergic receptor (Strader et al., 1991), κ-opioid receptor (Coward et al., 1998; Redfern et al., 1999; Vardy et al., 2015), and free fatty acid receptor 2 (Hudson et al., 2012). Although these methods are powerful for cell-specific activation of the downstream signaling pathways of the targeted GPCRs, they are unsuitable for investigating the physiological roles of receptors of interest that are endogenously expressed in the target cells, because the original ligand-binding properties of the receptors are altered.

Incorporation of a metal-binding site is another potential strategy for chemogenetic activation of POIs. Because of their strictly-defined coordination geometry, incorporation of coordinating amino acid residues at appropriate positions enables metal-induced conformational changes for activation of POIs (Yu et al., 2014; Ghanbarpour et al., 2019). In addition to successful examples of targeted soluble proteins, functional switching of GPCRs by metal coordination was previously demonstrated by Schwartz and co-workers. They introduced histidine (His) or cysteine (Cys) mutations into GPCRs such as tachykinin NK-1 receptor (Elling et al., 1995) and β2 adrenergic receptor (Elling et al., 1999) to yield metal-sensitive GPCRs. However, the mutations were introduced at the ligand-binding site, which resulted in reduced affinity to the endogenous ligand in most cases.

**FIGURE 1:**
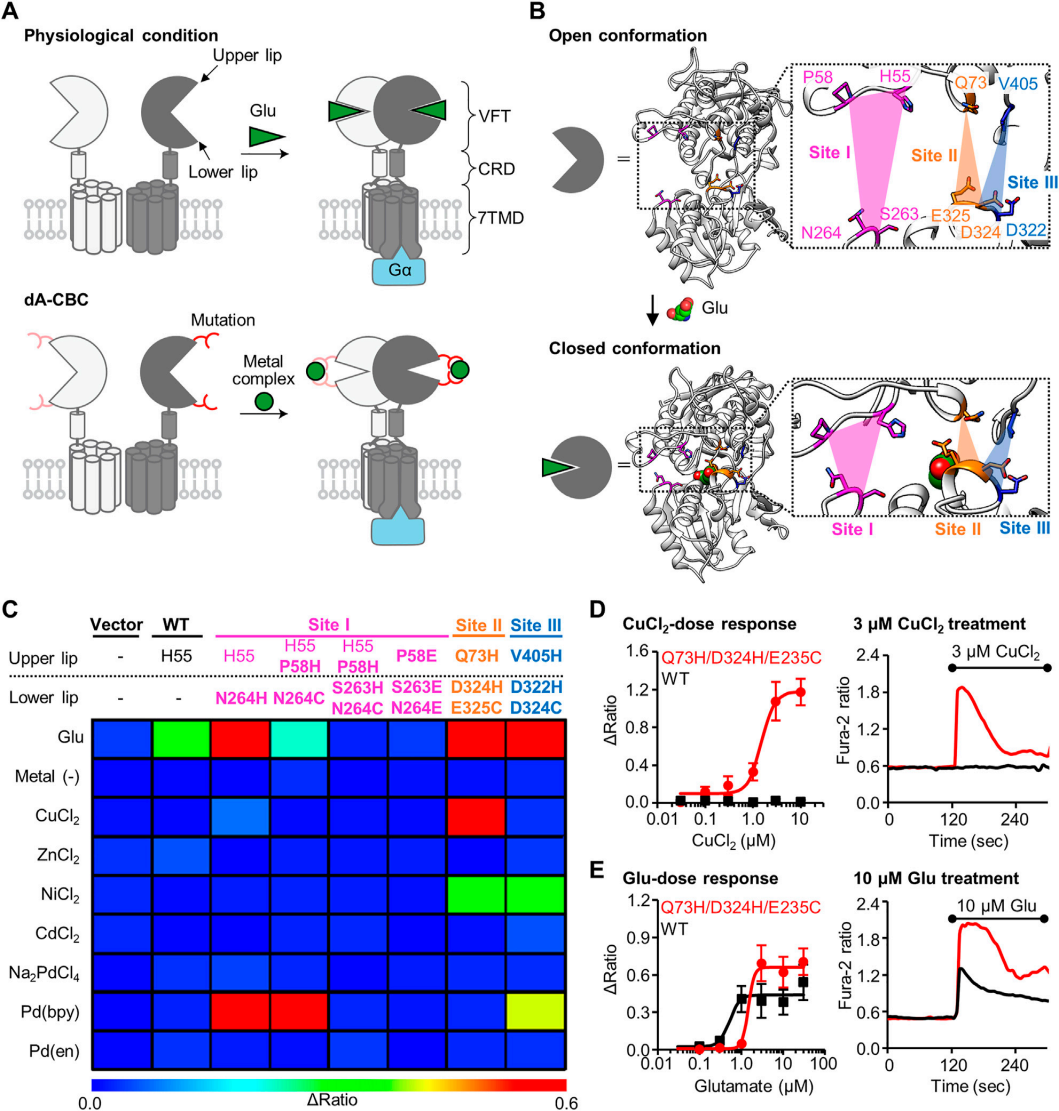
Screening of mGlu1 mutants with sensitivity to metal ion or complex. **(A)** Schematic illustration of activation of mGlu1 in physiological condition or in dA-CBC. In dA-CBC, addition of metal ion or complex induces the conformational change required for signal transduction. **(B)** Crystal structure of VFT domain of mGlu1 (PDB ID; 1EWT, 1EWK). Mutation sites are colored in magenta, orange, or blue, which are named Site I, Site II, or Site III, respectively. **(C)** Heat map of the primary screening. The heat map shows average Δratio values upon the addition of 10 μM glutamate or 3 μM each metal ion or complex. Mutation sites are described separately for upper or lower lip of VFT domain, and the mutation is shown in bold characters. See Supplementary Figure S2B for raw data. **(D)** Activation of the hit mutant by Cu^2+^. *Left*; dose dependency of Cu^2+^-induced activation of WT mGlu1 (black) or the Q73H/D324H/E325C mutant (red). The EC_50_ value of the mutant was 1.5 μM. (n = 11–15). *Right*; Representative traces of CuCl_2_ response as a function of time. The black bar represents the period when CuCl_2_ was added. **(E)** Activation of the hit mutant by glutamate (Glu). *Left*; Dose dependency of Glu-induced activation of WT mGlu1 (black) or Q73H/D324H/E325C mutant (red). The EC_50_ values were 0.52 or 1.4 μM for WT mGlu1 or the mutant, respectively. (n = 12–15). *Right*; Representative traces of glutamate response as a function of time. The black bar represents the period when glutamate was added. Data are represented as mean ± s.e.m.

To investigate the physiological roles of receptors of interest using chemogenetics, the target receptors should be mutated in a way that causes a specific response to the designed ligand without affecting the original receptor function. In this context, we have developed a chemogenetic method termed “direct activation *via* coordination-based chemogenetics (dA-CBC)” targeting metabotropic glutamate receptor 1 (mGlu1) by focusing on the structural changes upon glutamate binding (Kiyonaka et al., 2016; Kubota et al., 2019; Ojima et al., 2021). mGlu1, which belongs to class C GPCR, is composed of an extracellular ligand-binding domain called Venus Flytrap (VFT) domain, a cysteine rich domain (CRD), and a 7-transmembrane domain (7TMD) (Figure 1A). Glutamate binding to the VFT domain induces closure of the domain, and this signal is then transmitted to the 7TMD to cause receptor activation in mGlu1 (Kunishima et al., 2000; Tsuchiya et al., 2002). Inspired by the signal transduction induced by the structural changes, we introduced His mutations that cause metal-induced structural changes (Figures 1A,B). Through dA-CBC of mGlu1, His mutation (N264H) at the N264 position led to Pd-2,2′-bipyridine (Pd (bpy))-induced activation of the mGlu1 mutant (Ojima et al., 2021). In this case, Pd (bpy) coordinates endogenous H55 and N264H on the upper and lower lips of the VFT domain, respectively (Figure 1B). More importantly, the original ligand-binding properties were not affected in this method, which allowed us to prepare knock-in mice bearing the N264H mutation in the mGlu1 gene for chemogenetic regulation of endogenous mGlu1(N264H) (Ojima et al., 2021). dA-CBC has the potential for analyzing the physiological roles of mGlu1 endogenously expressed in the human brain. However, mGlu1 is expressed in various brain regions, such as the olfactory bulb, thalamus, hippocampus, and cerebellum (Lavreysen et al., 2004). Thus, orthogonal activation methods are necessary to simultaneously analyze the individual roles of mGlu1 in different brain regions.

In this paper, we identified two mGlu1 mutants that are selectively activated by Cu^2+^ or Zn^2+^ through structure-based design of the mutants and cell-based screening. Notably, these two mutants and the N264H mutant are activated in an orthogonal manner, allowing orthogonal activation of the three mGlu1 mutants by Pd (bpy), Cu^2+^, and Zn^2+^. Thus, these mGlu1 mutants could provide a potential platform for analyzing mGlu1 function in a cell-specific manner.

## Materials and Methods

### Construction of Expression Vector of mGlu1 Mutants

Site directed mutagenesis was performed using the Q5® Site-Directed Mutagenesis Kit (NEB) with pBluescript II SK (+) encoding rat mGlu1 by following the manufacture’s instruction. The cDNA of mGlu1 was subcloned into pCAGGS vector (Niwa et al., 1991) or pCDM vector to obtain the expression vectors. The pCDM vector were prepared from pcDNA3.1 (+) (Invitrogen), in which neomycin cassette was excised using *Pvu*II.

### Culture and Transfection of HEK293 Cells

HEK293 cells were maintained in Dulbecco’s modified Eagle’s medium (DMEM) with 100 unit ml^−1^ penicillin and 100 μg ml^−1^ streptomycin and 10% FBS (Sigma) at 37°C in a humidified atmosphere of 95% air and 5% CO_2_. HEK293 cells were transiently transfected with plasmids encoding WT mGlu 1, the mGlu1 mutants, or the control vector using Viafect (Promega) following the manufacture’s instruction. The cells were co-transfected with pEGFP-F (Clontech), pDsRed monomer-F (Clontech), or iRFP-670 (kindly gifted from Prof. Verkhusha) as transfection markers. For the culture of transfected cells, DMEM supplemented with 10% dialyzed FBS (Sigma) was used to decrease the cytotoxicity. The medium was exchanged 4 h after the transfection, and the cells were used for the experiments after 24–48 h.

### Fluorescent Ca^2+^ Imaging

The transfected HEK293 cells were seeded on glass coverslips (Matsunami) coated with poly-L-lysine solution (Sigma) and incubated for 4 h at 37°C in a humidified atmosphere of 95% air and 5% CO_2_. The calcium indicator Fura-2 AM (Dojindo) was loaded to the cells at 5 μM for 20–30 min. The imaging experiment was carried out in HBS buffer (20 mM HEPES pH 7.4, 107 mM NaCl, 6 mM KCl, 1.2 mM MgSO_4_, 2 mM CaCl_2_, 11.5 mM glucose). The fluorescence images and the Fura-2 ratio were measured using a fluorescence microscope (IX71, Olympus) equipped with a complementary metal-oxide semiconductor (CMOS) camera (ORCA-flash 4.0, Hamamatsu Photonics) under xenon-lamp illumination, and analyzed with a video imaging system (AQUACOSMOS, Hamamatsu Photonics) following the manufacture’s instruction. In imaging experiments, three different HEK293 cells transfected with one of the mGlu1 mutants were co-cultured on a glass coverslip, and each mutant was visually distinguished by the transfection markers. These three different cells were assayed simultaneously. The Δratio value was defined as the difference between the maximum ratio value after adding the reagent (metal ion or complex, or glutamate) and the average ratio before adding the reagent. The Δratio was fitted with KaleidaGraph to calculate the EC_50_ value using the equation: a + (b-a)/(1+(x/c)^d).

### Confocal Live Cell Imaging of Cell-Surface mGlu1 in HEK293 Cells

The HEK293 cells transfected with plasmids encoding WT mGlu1, the mGlu1 mutants, or control vector together with iRFP-670 as a transfection marker were seeded on glass bottom dishes coated with poly-L-lysine solution (Sigma) and incubated for 24 h at 37°C in a humidified atmosphere of 95% air and 5% CO_2_. After washing the cells with HBS buffer, 100 nM FITM-Cy3 in HBS was added to the dishes and incubated for 30 min at 16°C to suppress endocytosis. Confocal live imaging was performed using a confocal microscope (LSM900, Carl Zeiss) equipped with a 63×, numerical aperture (NA) = 1.4 oil-immersion objective. Fluorescence images were obtained by excitation at 561 or 640 nm derived from diode lasers. Fluorescence intensity from Cy3 on the cell surface was quantified from the line scans of iRFP-670-positive cells and calculated with subtraction of background.

### Statistical Analysis

All data are expressed as mean ± s.e.m. We accumulated the data for each condition from at least three independent experiments. We evaluated statistical significance with Student’s *t*-test or one-way ANOVA with Dunnet’s test. A value of *p* < 0.05 was considered significant.

## Results

### Design and Screening for Metal-Responsive mGlu1 Mutants

We previously reported that the conformational change of the VFT domain from an open to closed conformation by Pd (bpy) caused the selective activation of the mGlu1 (N264H) mutant. Notably, other metal ions (Cu^2+^, Zn^2+^, Ni^2+^, Cd^2+^, and Pd^2+^) failed to activate this mGlu1(N264H) mutant. Thus, identification of new mGlu1 mutants that are selectively activated by metal ions other than Pd (bpy) would allow orthogonal cell-specific activation of mGlu1 by transfecting either the mGlu1(N264H) mutant or the new mGlu1 mutants. To achieve this goal, we first designed a few more potential coordination sites around the N264 residue, which hereafter we call Site I (Figure 1B). Based on the mechanisms employed by Cu^2+^- or Zn^2+^-binding proteins such as azurin (Tian et al., 2016) or ZntR (Reyes-Caballero et al., 2011), a Cys residue was introduced in place of the His residue at N264 site. We designed the H55/P58H/N264C and H55/P58H/S263H/N264C mutants because Cu^2+^ or Zn^2+^ forms tridentate or tetradentate coordination in the metalloproteins (Figures 1B,C). In addition, in the cases of Ni^2+^- or Cd^2+^-binding proteins, acidic amino acids are sometimes used for the coordination of those metal ions (Trakhanov et al., 1998; Stegmann et al., 2010). Thus, we also designed P58E/S263E/N264E mutant (Figures 1B,C).

In addition to Site I, we focused on different regions in the VFT domain, termed Site II and Site III, as candidate coordination sites. At these sites, the distance between the upper and lower lips drastically changes upon domain closure (Figure 1B, Supplementary Figure S1). We designed the Q73H/D324H/E325C and V405H/D322H/D324C mutants from Site II and Site III, respectively, for metal ion-induced receptor activation (Figures 1B,C). Note that the mutation residues selected here do not participate in glutamate binding (Kunishima et al., 2000), so we expected that the designed mutants would maintain the original activity of mGlu1. Collectively, we performed primary screening of a total of five mutants from Site I through Site III for obtaining metal-responsive mGlu1 mutants.

Fluorescent Ca^2+^ imaging was performed to check whether these mutants are activated by metal ions or complexes (Supplementary Figure S2A). Because mGlu1 is a Gq-coupled GPCR, its activation causes elevated intracellular Ca^2+^ concentrations ([Ca^2+^]_i_) *via* phospholipase C (PLC) activation. Each mutant was transiently transfected into HEK293 cells, and the [Ca^2+^]_i_ changes upon the addition of each metal-ion or complex were monitored by a fluorescent Ca^2+^ indicator, Fura-2. As control experiments, the vector control, wild-type (WT) mGlu1, and the previously reported Pd (bpy)-selective N264H mutant were also examined. As expected on the basis of our prior report, metal ions and metal complexes failed to activate WT mGlu1, whereas Pd (bpy) selectively activated the N264H mutant (Figure 1C). We subsequently examined the effects of the metal ions or complexes on the newly designed mGlu1 mutants. Focusing on the mGlu1 mutants from Site I, although the H55/P58H/N264C mutant was activated by Pd (bpy), none of the three newly designed mutants, including the H55/P58H/N264C mutant, were activated by other metal ions. (Figure 1C, Supplementary Figure S2B). Interestingly, the V405H/D322H/D324C mutant from Site III showed sensitivity to Ni^2+^. However, this mutant was also activated by Pd (bpy).

In contrast to these mutants from Site I and III, the Q73H/D324H/E325C mutant from Site II was strongly activated by Cu^2+^ while also showing moderate sensitivity to Ni^2+^ (Figure 1C). Notably, the Q73H/D324H/E325C mutant was not activated by Pd (bpy), hence allowing orthogonal activation of this mutant and the N264H mutant by Cu^2+^ and Pd (bpy) respectively. The activation of the Q73H/D324H/E325C mutant by Cu^2+^ was further validated by the dose-dependent response of Cu^2+^ in the fluorescent Ca^2+^ imaging. As shown in Figure 1D, the EC_50_ value was 1.5 µM, while WT mGlu1 was not activated at that concentration of Cu^2+^ at all. Besides, the EC_50_ value of glutamate (1.4 µM) for the mutant slightly shifted to a higher concentration but was comparable to that for the WT mGlu1 (0.52 µM), indicating that this mutant preserved the original ligand-binding properties of mGlu1 (Figure 1E). Therefore, we selected the Q73H/D324H/E325C mutant as a Cu^2+^-responsive mGlu1 mutant from the primary screening.

### Optimization and Characterization of Cu^2+^-Responsive mGlu1 Mutant

**FIGURE 2:**
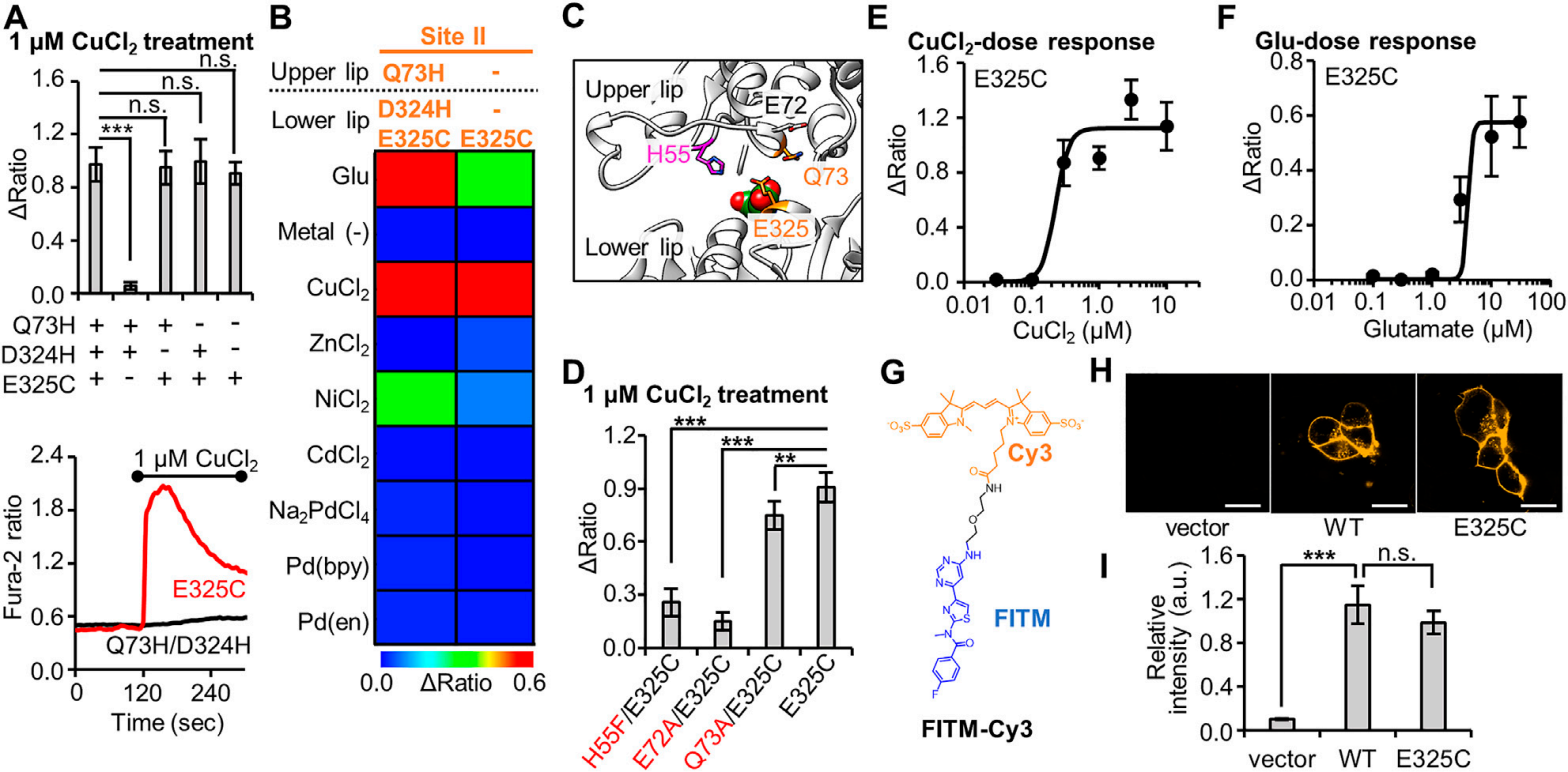
Optimization of Cu^2+^-responsive mGlu1 mutant and its characterization. **(A)** Mutation study to confirm the importance of His- and Cys-incorporation at the mutation sites. *Upper*; the average Δratio values for Q73H/D324H/E325C, Q73H/D324H, Q73H/E325C, D324H/E325C, or E325C mutant upon the treatment of 1 μM CuCl_2_. (n = 12–20). *Lower*; representative traces from E325C or Q73H/D324H mutant. The black bar represents the period when CuCl_2_ was added. **(B)** Heat map showing the metal selectivity of the newly identified Cu^2+^-responsive E325C mutant. The map shows the average Δratio values upon the addition of 10 μM glutamate or 3 μM metal ion or complex. See Supplementary Figure S3 for raw data. **(C)** Candidate residues for the coordination of Cu^2+^ with E325C in the VFT domain (PDB ID; 1EWK). **(D)** Mutation study to identify the coordination residues. The average Δratio values upon the treatment of 1 μM CuCl_2_ are shown. (n = 12–20). **(E)** Activation of the mGlu1 E325C mutant by Cu^2+^. The EC_50_ value for mGlu1 E325C mutant was 0.22 μM. (n = 12–20). **(F)** Activation of the mGlu1 E325C mutant by glutamate (Glu). The EC_50_ value of the mutant was 4.0 μM. (n = 12–20). **(G)** Chemical structure of FITM-Cy3. **(H)** Evaluation of the expression and distribution of mGlu1 mutant on the cell surface using FITM-Cy3. The representative confocal images are shown. The scale bar shows 20 μm. **(I)** Quantification of signal intensity from FITM-Cy3. (n = 20–31). Data are presented as mean ± s.e.m. ***Significant difference (*p* < 0.001, One-way ANOVA with Dunnet’s test). **Significant difference (*p* < 0.01). n.s, not significant (*p* > 0.05).

We proceeded to evaluate the Q73H/D324H/E325C mutant in further detail. To confirm the importance of the mutated His or Cys residues, we designed the Q73H/D324H, Q73H/E325C, and D324H/E325C mutants, where one of the three mutated residues from the Q73H/D324H/E325C mutant was substituted back with the original amino acid residue. As expected, fluorescent Ca^2+^ imaging revealed a loss in sensitivity to Cu^2+^ for the Q73H/D324H mutant (Figure 2A). However, unexpectedly, prominent Cu^2+^-induced responses were observed in the case of the Q73H/E325C and D324H/E325C mutants. Since all Cu^2+^-responsive mutants shared the same E325C mutation, we next evaluated the Cu^2+^-induced response of the single mutant, mGlu1 (E325C). As shown in Figure 2A, the Cu^2+^-induced response was maintained in the single mutant, suggesting that the E325C mutation alone is sufficient for the activation by Cu^2+^. In addition, the E325C mutant showed a high selectivity to Cu^2+^ (Figure 2B, Supplementary Figure S3). Considering that the activation mechanism of mGlu1 requires the VFT domain closure, the coordination partner should be found in the upper lip of that domain. According to the crystal structure of the closed conformation of the VFT domain (PDB ID; 1EWK), endogenous residues such as H55, E72, and Q73 are candidate residues for Cu^2+^ coordination in collaboration with the E325C mutation (Figure 2C). To check this possibility, these candidate residues were substituted with phenylalanine or alanine, and the Cu^2+^-induced responses of the H55F/E325C, E72A/E325C, and Q73A/E325C mutants were subsequently examined. As shown in Figure 2D, the sensitivity to Cu^2+^ was slightly affected for the Q73A/E325C mutant. In contrast, the Cu^2+^-induced responses drastically decreased for the H55F/E325C and E72A/E325C mutants, suggesting that H55 and E72 could be potential coordination partners of E325C for Cu^2+^-induced activation.

Next, we performed a functional characterization of the newly identified E325C mutant. The dose-dependency of Cu^2+^-induced responses revealed that the EC_50_ value of Cu^2+^ for this mutant was 0.22 µM (Figure 2E), which is lower than that for the Q73H/D324H/E325C mutant (Figure 1D). The EC_50_ value of glutamate to induce activation of the E325C mutant was 4.0 µM (Figure 2F). As was the case with the Q73H/D324H/E325C mutant, the EC_50_ value of glutamate for the E325C mutant slightly increased but was still on the same order of magnitude as that for the WT mGlu1. We also assessed the distribution and expression level of the E325C mutant under live-cell conditions. Here, we used a synthetic fluorescent probe named FITM-Cy3, which is a Cy3-conjugated FITM ligand (Figure 2G). FITM is a negative allosteric modulator (NAM) that binds to the 7TMD of mGlu1 (Supplementary Figure S4) (Wu et al., 2014), and FITM-Cy3 binds to cell-surface mGlu1 with high affinity (*K*_d_ = 6.8 nM) (Ojima et al., 2021). Thus, the distribution of the mGlu1 mutant can be estimated quantitatively using FITM-Cy3. As shown in Figure 2H, confocal live imaging of HEK293 cells transfected with either the WT mGlu1 or the E325C mutant revealed the clear localization of the probe on the cell surface, while the fluorescent signal was hardly detectable in vector-transfected cells. Importantly, the fluorescent intensity was not significantly different (*p* > 0.05) between the WT mGlu1 and the E325C mutant (Figure 2I), suggesting that the localization and expression level of the E325C mutant was unaffected by the mutation. In summary, through a precise analysis of the hit mutant obtained from the primary screening, we concluded the E325C mutant is a Cu^2+^-responsive mGlu1 mutant.

### Identification of Zn^2+^-Responsive mGlu1 Mutant

In metal-binding proteins, the coordination geometry has essential roles for the function or metal-selectivity. For instance, the metal ion selectivity of azurin, a Cu^2+^-binding protein, is affected by the geometry of the coordinating residues (Nar et al., 1992). As described above (see Figure 1C), we revealed that the V405H/D322H/D324C mutant from Site III was activated by both Ni^2+^ and Pd (bpy) in the primary screening. As a result, this mutant cannot be applied for orthogonal activation in conjunction with the Pd (bpy)-responsive mutant. Based on examples of prior successes such as that of azurin, we proceeded to perform secondary screening on the V405H/D323H/D324C (HHC) mutant by changing the geometry of the mutated residues.

**FIGURE 3:**
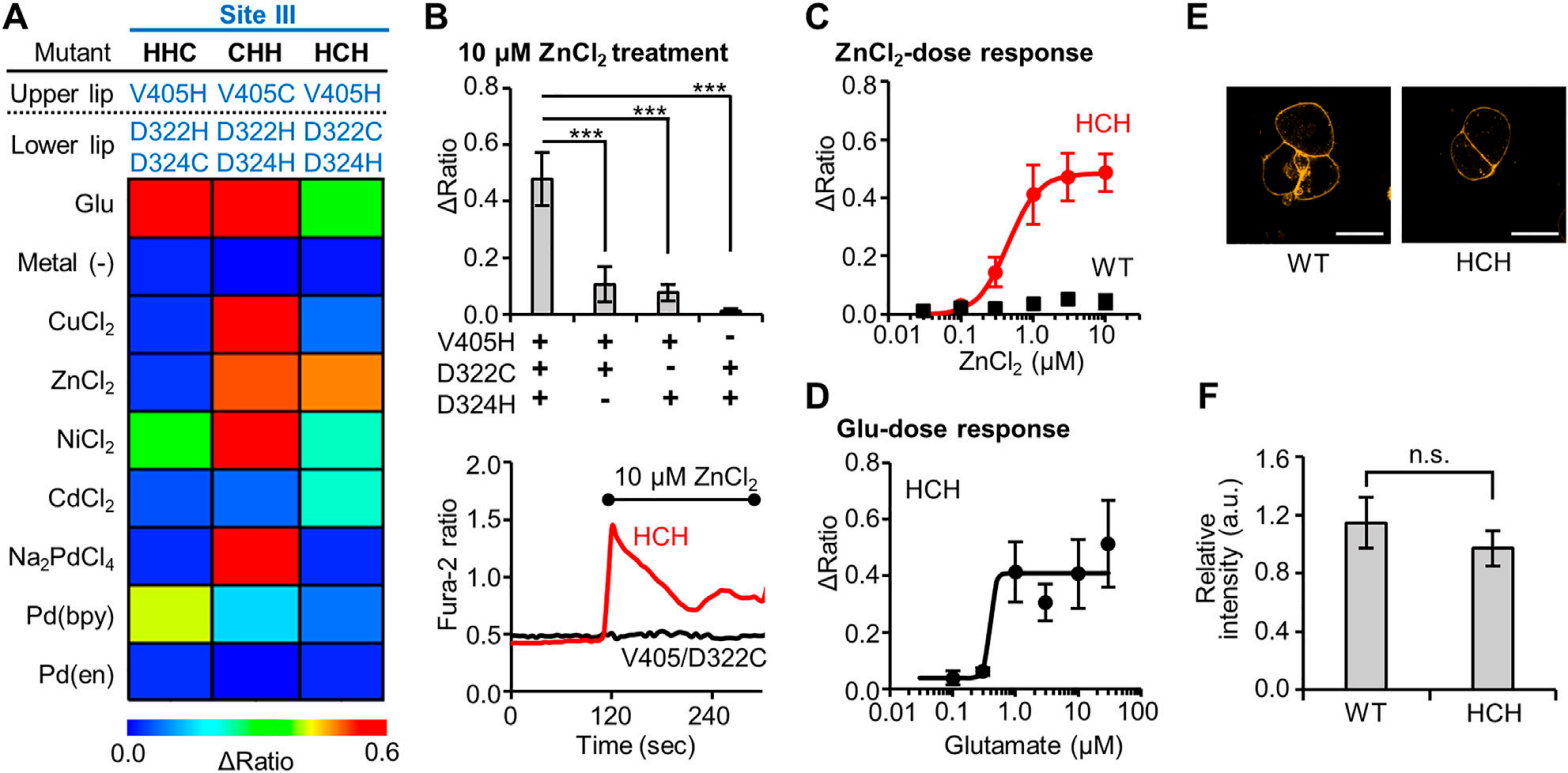
Identification of Zn^2+^-responsive mGlu1 mutant and its characterization. **(A)** Heat map of the secondary screening. The map shows the average Δratio values upon the addition of 10 μM glutamate or 3 μM metal ion or complex. See Supplementary Figure S5 for raw data. **(B)** Mutation study to confirm the Zn^2+^-coordination at the mutation sites. *Upper*; The Δratio values for the HCH, V405H/D322C, V405H/D324H, and D322C/D324H mutants. *Lower*; representative traces of the cells from two mutants. The black bar shows the period when ZnCl_2_ was added. (n = 12–20). **(C)** Activation of WT mGlu1 (black) or the HCH mutant (red) by Zn^2+^. The EC_50_ value for the mutant was 0.44 μM. (n = 11–20). **(D)** Activation of the HCH mutant by glutamate (Glu). The EC_50_ value of the mutant was 0.39 μM. (n = 11–20). **(E)** Evaluation of the expression and distribution of mGlu1 mutant on the cell surface using FITM-Cy3. The representative confocal images are shown. The scale bar shows 20 μm. (n = 50–70). **(F)** Quantification of signal intensity from FITM-Cy3. Data are presented as mean ± s.e.m. ***Significant difference (*p* < 0.001, One-way ANOVA with Dunnet’s test). n.s, not significant (*p* > 0.05, Student’s *t*-test.).

In the secondary screening, we designed two more mutants, V405C/D322H/D324H (CHH) and V405H/D322C/D324H (HCH), where we altered the position of the Cys residue without changing the mutation sites themselves. Similar to the characterization of the primary screening, metal-induced cellular responses were again evaluated using fluorescent Ca^2+^ imaging. As illustrated in the heat map in Figure 3A, the two newly designed mutants (CHH and HCH) showed drastically different metal-selectivity, compared with the original HHC mutant. The CHH mutant showed a broad selectivity since it was strongly activated by several metal ions such as Cu^2+^, Zn^2+^, Ni^2+^, and Pd^2+^. In contrast, the HCH mutant was activated strongly by Zn^2+^, moderately by Ni^2+^ or Cd^2+^, and not activated at all by either Cu^2+^ or Pd (bpy) (Figure 3A, Supplementary Figure S5). Therefore, the HCH mutant has the potential to be another orthogonal mutant for use along with the Pd (bpy)-responsive N264H mutant and the Cu^2+^-responsive E325C mutant.

Because Zn^2+^ strongly activated this HCH mutant, the Zn^2+^-coordination was further analyzed. First, we evaluated whether all three mutations were necessary for the Zn^2+^-coordination. For this purpose, three mutants, V405H/D322C, V405H/D324H, and D322C/D324H were designed, where each of the three mutated residues in the HCH mutant was individually changed back to the original residue. As shown in Figure 3B, the Zn^2+^-induced responses were significantly impaired in the three mutants, suggesting that all three mutations were required for the Zn^2+^-induced activation of the mGlu1 mutant.

We then further studied the Zn^2+^-response of this mutant in detail. The dose-dependent response of Zn^2+^ determined the EC_50_ value to be 0.44 µM for the mutant, while the WT mGlu1 was not activated at all at this Zn^2+^ concentration (Figure 3C). To verify whether the original ligand-binding property of mGlu1 is preserved in this mutant, the dose-dependent glutamate response was also measured. As shown in Figure 3D, the EC_50_ value of glutamate for the activation of this mutant was 0.39 µM, which was comparable to that of the WT mGlu1 (0.52 µM, Figure 1E). In addition, confocal live imaging using FITM-Cy3 revealed that the distribution and the expression level of the mGlu1 mutant were not affected by the introduction of these mutations (Figures 3E,F). In summary, our secondary screening identified the HCH mutant as a Zn^2+^-responsive mGlu1 mutant which was insensitive to Pd (bpy) and Cu^2+^.

### Cell-specific Activation Utilizing Mutually Orthogonal mGlu1 Mutants

Through the primary and secondary screening, we identified two more mutants in addition to the Pd (bpy)-responsive N264H mutant: the Cu^2+^-responsive E325C and Zn^2+^-responsive HCH mutants. Notably, these three mutants have sufficient metal-selectivity among Cu^2+^, Zn^2+^, and Pd (bpy). Namely, N264H was not activated by Cu^2+^ or Zn^2+^, E325C was not activated by Zn^2+^ or Pd (bpy), and HCH was not activated by Cu^2+^ or Pd (bpy) (Supplementary Figure S6). Besides, the metal-induced activations were repeatedly observed after washing out the bound metals for 2 min, suggesting a minutes-order reversibility of the activation (Supplementary Figure S7). However, the activation of the E325C mutant by Cu^2+^ or the HCH mutant by Zn^2+^ was impaired in the co-presence of Pd (bpy) or Cu^2+^, respectively (Supplementary Figure S8).

**FIGURE 4:**
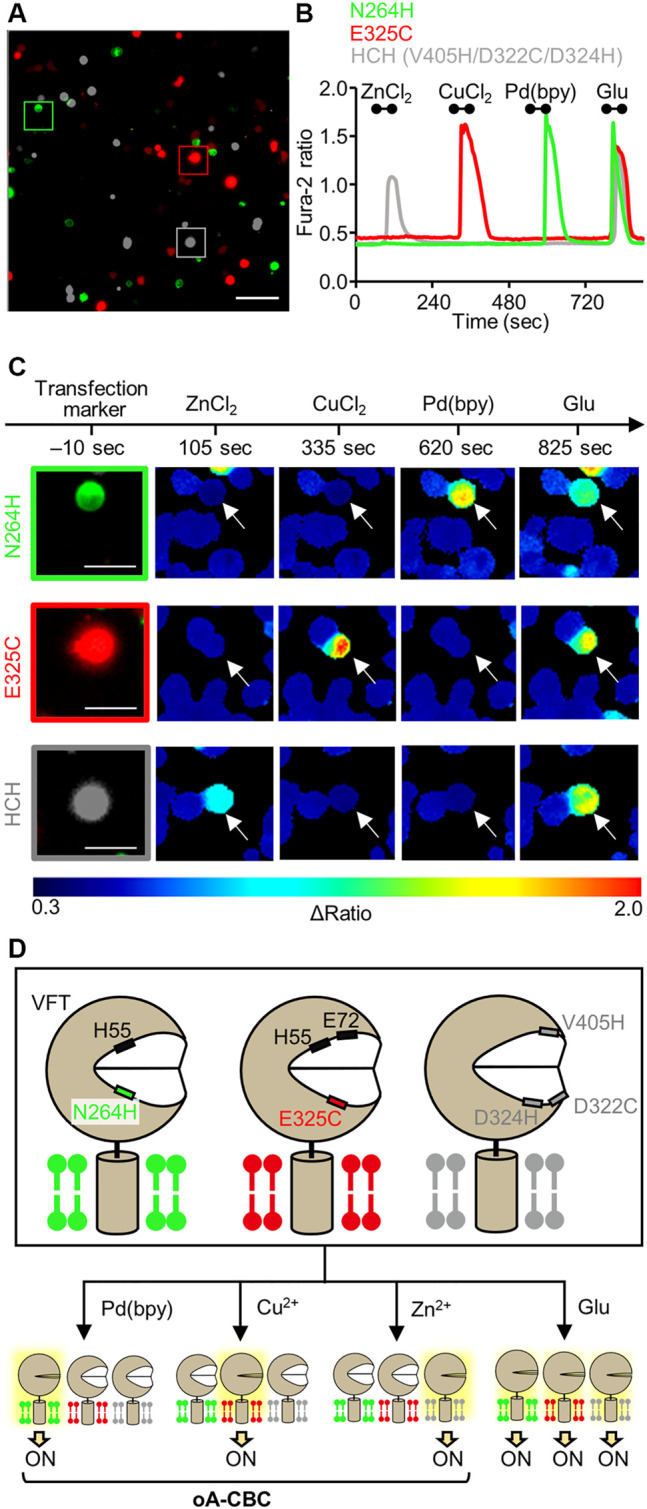
Orthogonal activation of three mGlu1 mutants with Pd(bpy), Cu^2+^, and Zn^2+^. **(A)** Merged fluorescent images of HEK293 cells transfected with N264H (green), E325C (red), or HCH mutant (gray). The scale bar shows 100 μm. **(B)** Orthogonal activation of the N264H, E325C, HCH mutant by 3 μM Pd (bpy), 1 μM CuCl_2_, or 3 μM ZnCl_2_, respectively. 3 μM glutamate (Glu) was added after the metal ions or complex. The traces of Δratio values as a function of time for the representative cells marked in (A) are shown. The black bars inside the figure show the period when each reagent was added. **(C)** The transfection markers and Δratio images of the cells expressing each mutant. The white arrows show the representative cells marked in (A). The scale bars show 25 μm. **(D)** Schematic illustration of oA-CBC system. Mutations in VFT domain produced three types of mutants orthogonally sensitized to Pd (bpy), Cu^2+^, and Zn^2+^. These mutants allow the cell-specific activation by Pd (bpy), Cu^2+^, and Zn^2+^, while all of three mutants are activated by Glu, because they keep the original ligand binding property.

Considering these properties of the three mutants, we demonstrated cell-specific mGlu1 activation. Here, we prepared a model experiment for the cell-specific regulation of mGlu1, where HEK293 cells were transfected with a plasmid encoding either the N264H, E325C, or HCH mutant. In this experiment, we regarded each of these transfected cells as different cell types. To visually distinguish these cells, the transfection markers EGFP, DsRed-monomer, and iRFP-670 were co-expressed with the N264H, E325C, and HCH mutant, respectively. After 48 h of transfection, these three types of HEK293 cells were mixed and seeded on a single glass coverslip (Figure 4A). Given the impairment on the metal-induced activation of the E325C or HCH mutant in the co-presence of Pd (bpy) or Cu^2+^, respectively, ZnCl_2_, CuCl_2_, and Pd (bpy) were sequentially applied for 60 s onto the glass coverslip *via* perfusion in this order. As shown in Figures 4B,C, the fluorescent Ca^2+^ imaging revealed that each metal ion or complex selectively activated the cells that expressed its corresponding mutant. Notably, the glutamate-induced responses were intact after metal-induced activation, suggesting that mGlu1 activity was not affected after metal-induced activation. These results indicate that the HEK293 cells that expressed the three different types of mGlu1 mutants were selectively activated by either Zn^2+^, Cu^2+^, or Pd (bpy), thus demonstrating an orthogonal trio for mGlu1 activation. We termed this method orthogonal activation *via* coordination-based chemogenetics (oA-CBC) of mGlu1 (Figure 4D).

## Discussion

In this study, three mutually orthogonal mutants for the dA-CBC system were developed. Specifically, a N264H mutant sensitized to Pd (bpy), a E325C mutant sensitized to Cu^2+^, and a HCH mutant sensitized to Zn^2+^ were identified and characterized. Fluorescent Ca^2+^ imaging using HEK293 cells demonstrated that three kinds of metal ions or complexes orthogonally activated the cells expressing a corresponding mGlu1 mutant. This system was termed as the oA-CBC system. Although our current study was limited to demonstrating this orthogonal activation in HEK293 cells, the oA-CBC system has the potential to analyze mGlu1 function in several different human brain regions simultaneously using tissue-selective promoters in the brain, given that mGlu1 is expressed in the olfactory bulb, thalamus, hippocampus, and cerebellum in the brain (Lavreysen et al., 2004).

The mGlu family comprises eight subtypes (mGlu1–8) and is classified into three groups (group I–III) based on the amino-acid sequence and transducing signal properties of each subtype (Niswender and Conn, 2010). mGlu1 and mGlu5 are both “group I” because of their closely homologous sequences that couple to Gq-proteins. Although both are predominantly expressed in the brain, their distribution is different. Unlike mGlu1, mGlu5 is expressed in the caudate-putamen, lateral septum, and cortex in the brain (Romano et al., 1995). Although both subtypes are expressed in the hippocampus, previous studies revealed that the functional contributions of each subtype are different in the region (Neyman and Manahan-Vaughan, 2008). Group I metabotropic glutamate receptor agonists such as 3,5-dihydroxyphenylglycine (DHPG) are frequently used to analyze the physiological roles of these receptors (Fitzjohn et al., 1999). However, this agonist cannot discriminate mGlu1 from mGlu5 because of the high sequence homology. Thus, it is challenging to independently activate and analyze mGlu1 or mGlu5 expressed in the same region of the brain. Therefore, potential future work could be to apply the oA-CBC method to mGlu5, thus creating an orthogonal pair of mGlu1 and mGlu5 mutants. The mutants could be activated by two different metal ions or complexes and utilized for the subtype-selective activation of mGlu proteins.

Another future application of our oA-CBC method is to construct an artificial signal-transducing system using three different metal ions or complexes as its input. Since mGlu1 is coupled to Gq-protein, the activation of mGlu1 by a metal ion or complex leads to the elevation of [Ca^2+^]_i_ *via* PLC activation. However, the other mGlu proteins in groups II or III coupled with Gi/o-protein decreases the intracellular cAMP level by inhibiting adenylyl cyclase activity (Niswender and Conn, 2010). The oA-CBC method can also be applied to other types of mGlu proteins to artificially hijack different types of G-protein signals in an orthogonal way.

## Data Availability

The original contributions presented in the study are included in the article/Supplementary Material, further inquiries can be directed to the corresponding author.
