# Supplementary material for: Orthogonal Activation of Metabotropic Glutamate Receptor Using Coordination Chemogenetics
Source: Front Chem. 2022 Jan 14;9:825669. doi: 10.3389/fchem.2021.825669 (PMC8795677; doi:10.3389/fchem.2021.825669)
Supplement: Supplementary file 1 [file DataSheet1.pdf]

Supplementary Material

Supplementary Figures

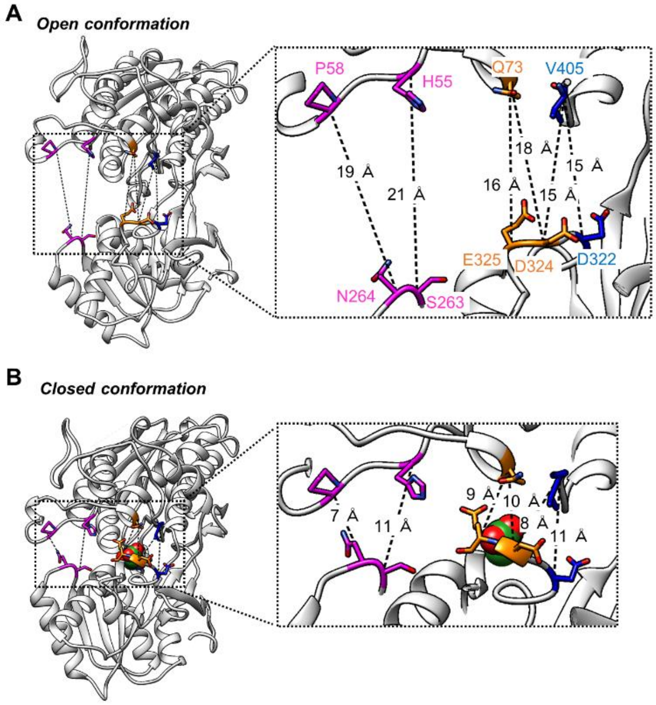

Supplementary Figure S1. Distances between Cα atoms from the residues of mutation sites on the upper lip and the lower lip. Distances between P58 – N264, H55 – S263, Q73 – D324, Q73 – E325, V405 – D322, and V405 – D324 are shown in dotted lines in open conformation **(A)** or in closed conformation **(B)** of VFT domain.

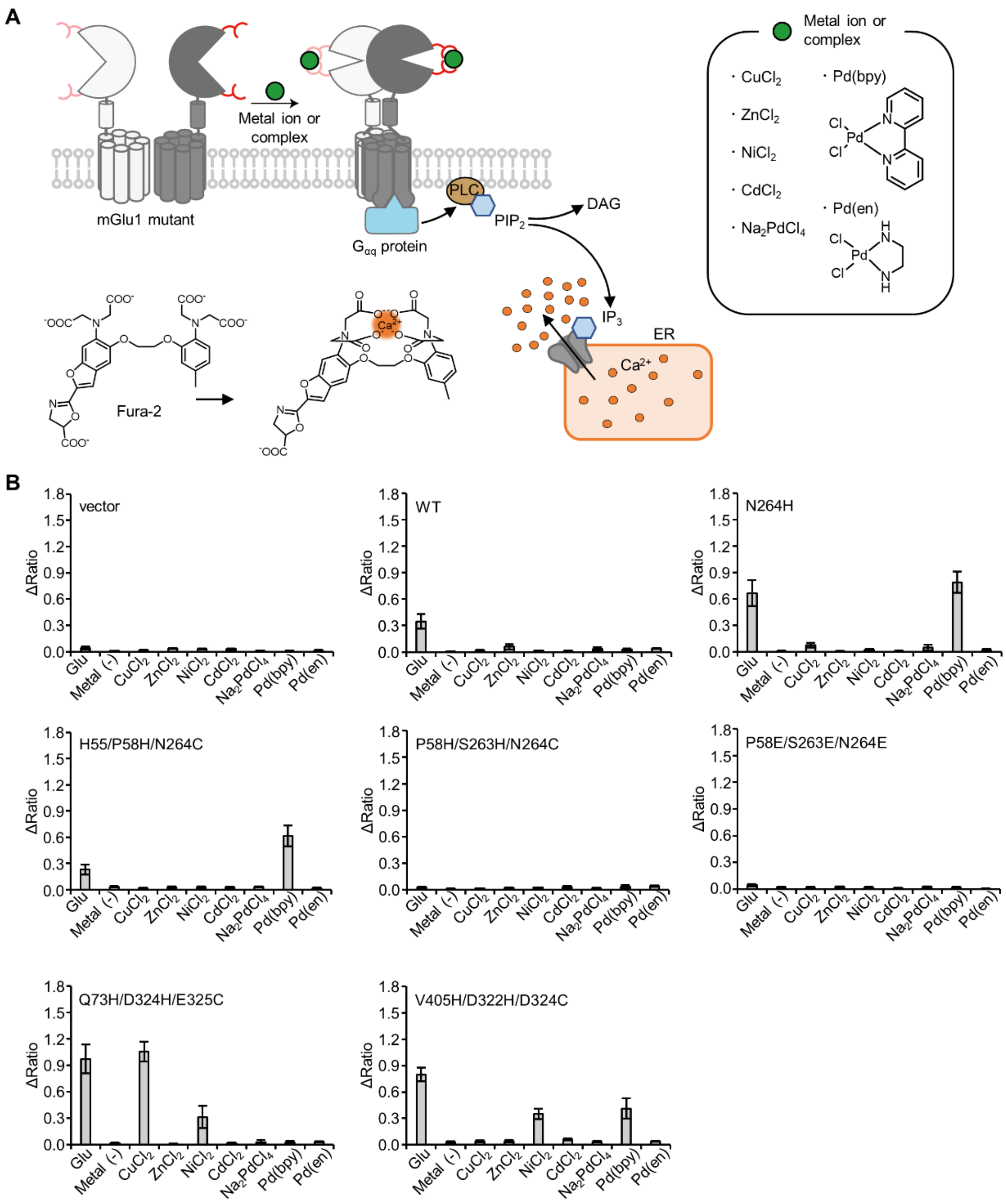

Supplementary Figure S2. Screening of mGlu1 mutants sensitized to metal ions or complexes. (A) Schematic illustration of fluorescent Ca^{2+} imaging. Activation of Gq-coupled mGlu1 leads to the increase in intracellular Ca^{2+} concentration ($[Ca^{2+}]_i$) through the Ca^{2+} release from endoplasmic reticulum (ER). The $[Ca^{2+}]_i$ is determined by Fura-2 ratio (340/380 nm). **(B)** Averaged Δratio values induced by 3 μM metal ion or complex, or 10 μM glutamate are shown. (n = 12–20). Data are presented as mean ± s.e.m.

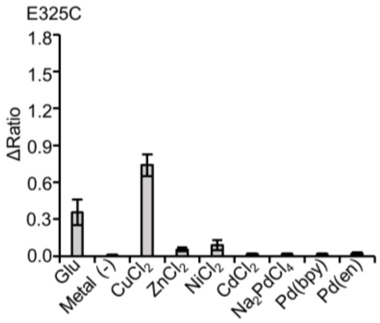

Supplementary Figure S3. Metal selectivity of the E325C mutant. Average Δratio values induced by 3 μM metal ion or complex, or 10 μM glutamate are shown. (n = 10–20). Data are presented as mean ± s.e.m.

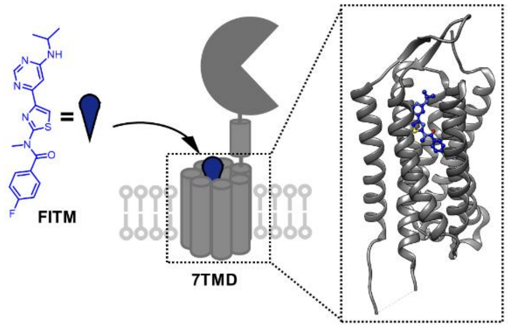

Supplementary Figure S4. Binding mode of FITM. FITM is a NAM of mGlu1, which binds to 7TMD of mGlu1. The crystal structure shows the binding mode of FITM (PDB ID; 4OR2).

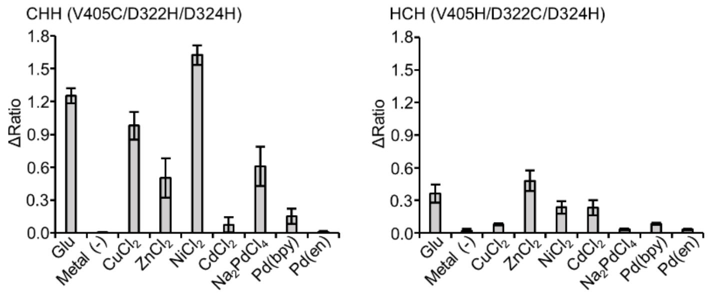

Supplementary Figure S5. The secondary screening of mGlu1 mutants aiming at three-orthogonal activation. Average Δratio values induced by 3 μM metal ion or complex, or 10 μM glutamate are shown. (n = 11–20). Data are presented as mean ± s.e.m.

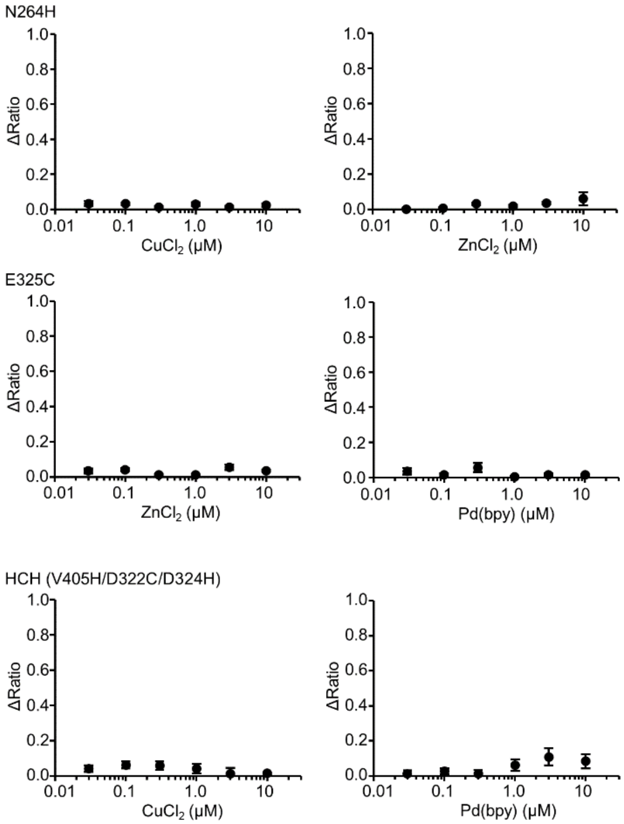

Supplementary Figure S6. Confirmation of selective activation of each mutant by metal ions or complex. Average Δratio values upon the addition of each metal ions or complex are shown. Data are presented as mean ± s.e.m. (n = 12–20). See also Ref. (Ojima et al., 2021) for the N264H mutant by Pd(bpy), Figure 2D for the E325C mutant by Cu^{2+}, and Figure 3C for the HCH mutant by Zn^{2+}.

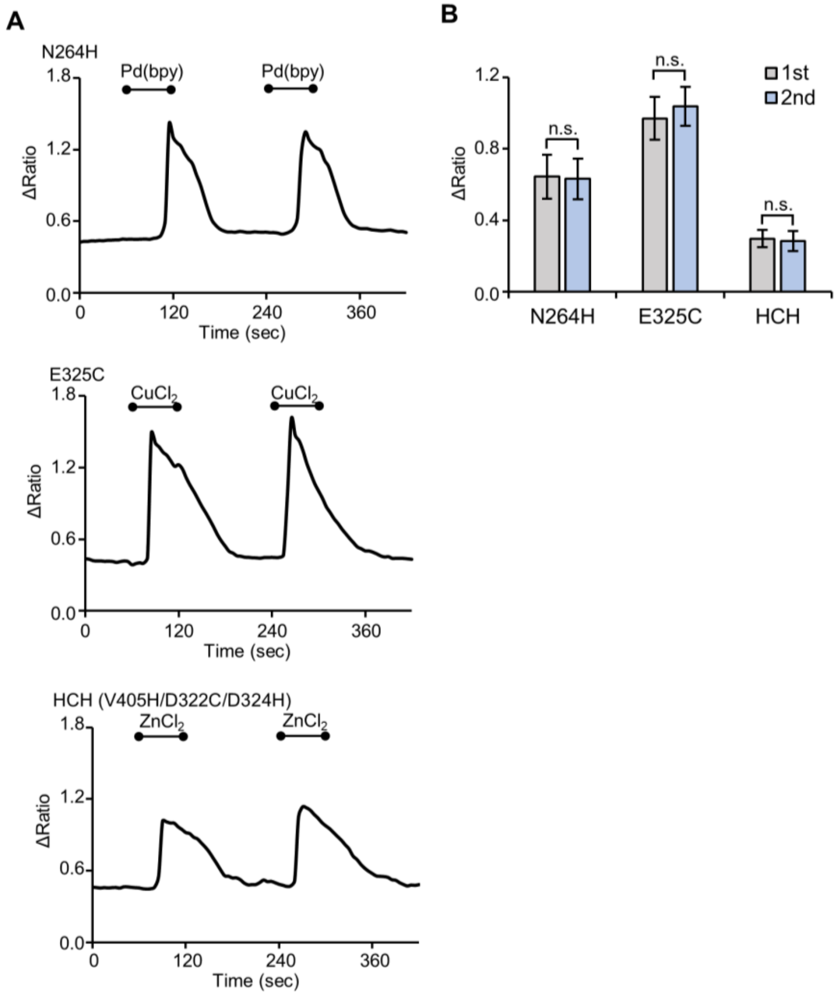

Supplementary Figure S7. Reversibility in the activation of each mutant by metal ions or complex. (A) Representative traces of repeated activation of each mutant. The black bars inside the figures show the period when each reagent was added. [Pd(bpy)] = 3 μM, [$CuCl_2$] = 1 μM, [$ZnCl_2$] = 3 μM. **(B)** Average Δratio values obtained from the first and second activations. Data are presented as mean ± s.e.m. (n = 12–15). n.s., not significant ($P > 0.05$, Student's *t*-test).

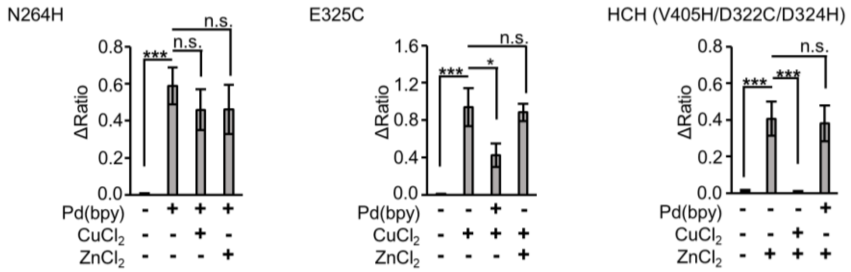

Supplementary Figure S8. Activation of each mutant in the presence of the other metal ion or complex. ΔRatio values upon the addition of Pd(bpy), $CuCl_2$, and $ZnCl_2$ are shown. [Pd(bpy)] = 3 μM, [$CuCl_2$] = 1 μM, [$ZnCl_2$] = 3 μM. Co-presence of Cu^{2+} or Zn^{2+} did not affect the activation of the N264H mutant by Pd(bpy), while co-presence of Pd(bpy) impaired the activation of the E325C mutant by Cu^{2+}, and co-presence of Cu^{2+} impaired the activation of the HCH mutant by Zn^{2+}. Data are presented as mean ± s.e.m. (n = 11–20). ***Significant difference ($P < 0.001$, One-way ANOVA with Dunnet's test). *Significant difference ($P < 0.05$). n.s., not significant ($P > 0.05$).
